# Understanding symptom contribution to sex inequality in bladder and renal cancer stage at diagnosis

**DOI:** 10.1002/bco2.360

**Published:** 2024-04-19

**Authors:** Yin Zhou, Georgios Lyratzopoulos, Prabhakar Rajan, Fiona M. Walter, Jianhua Wu

**Affiliations:** ^1^ Wolfson Institute of Population Health Queen Mary University of London London UK; ^2^ Institute of Epidemiology and Health Care University College London London UK; ^3^ Barts Cancer Institute, Cancer Research UK City of London Centre Queen Mary University of London London UK; ^4^ Department of Urology, Barts Health NHS Trust The Royal London Hospital London UK

**Keywords:** bladder cancer, cancer stage, early diagnosis, renal cancer, sex inequality, symptomatic presentation

## Abstract

**Background:**

Understanding sex‐specific factors contributing to advanced‐stage diagnosis can guide interventions to reduce sex inequality in patients with urological cancers.

**Method:**

We used linked primary care and cancer registry data to examine associations between symptoms and advanced‐stage in 1151 bladder cancer and 440 renal cancer patients diagnosed between January 2012 and December 2015 in England. We performed logistic regression, adjusting for sex, age, deprivation and routes to diagnosis, including interaction terms between symptoms and sex and symptoms and age.

**Results:**

Female sex (OR vs. men 1.89 [1.28–2.79]; *p* = 0.001) and patients presenting with urinary tract infections (OR 2.22 [1.34–3.69]) and abdominal symptoms (OR 2.19 [1.30–3.70]) were associated with increased odds of advanced‐stage bladder cancer (vs. haematuria, *p* = 0.016 for both). Women with haematuria and men with abdominal symptoms (compared with the opposite sex with the same presenting symptom) were more likely to have advanced‐stage bladder cancer. Neither sex nor symptom associations were observed for renal cancer.

**Conclusion:**

Non‐haematuria symptoms are associated with higher risk of advanced‐stage bladder cancer. Greater risk of advanced‐stage bladder cancer in women may reflect biological differences in haematuria onset and sex differences during diagnostic process. Identifying higher risk women with haematuria may reduce sex inequalities in bladder cancer outcomes.

## 1 BACKGROUND

Bladder and renal cancer are among the top 10 most common cancers in Europe.^1^ Variations in timeliness of cancer diagnosis and survival outcomes exist, especially for bladder cancer.^2^ Hence, a greater understanding of factors that affect early‐stage diagnosis is crucial to improving clinical outcomes and patient experience.^3^, ^4^

Women were more likely to have delayed investigations, referrals and diagnosis, as well as worse cancer survival outcomes compared with men.^2^, ^5^, ^6^ The sex disparity in survival is greatest in bladder cancer compared to all other common and rarer cancers; a smaller sex disparity in survival exist for renal cancer.^6^ Many research, clinical and policy efforts to improve bladder and renal cancer outcomes have aimed to improve public awareness of haematuria, the main alarm symptom associated with these cancers, and access to diagnostics for haematuria evaluation. However, efforts should also focus on other more common symptoms,^7^ as less than 20% of symptomatic renal cancer patients present with haematuria,^8^ and a significant minority of bladder cancer patients present with lower risk symptoms such as recurrent urinary tract infections (UTIs) and non‐visible haematuria. These are associated with increased risk of missed diagnostic opportunities.^9^

It is not yet known whether sex disparities in stage at diagnosis relate to differences in symptoms. Hence, understanding the associations between presenting symptoms and stage of diagnosis may shed light on why the sex disparity exists in diagnostic timeliness and outcomes and barriers to early‐stage diagnosis. Improved knowledge will allow the development of interventions to improve early diagnosis and reduce survival inequalities.

Therefore, we examine the associations between common symptoms of possible bladder and renal cancer and stage of cancer diagnosis, and whether and to what extent presenting symptoms might explain the sex differences in cancer stage at diagnosis seen in patients with these two cancers.

## 2 METHOD

### 2.1 Data and cohort

We used linked Clinical Practice Research Datalink (CPRD) GOLD, National Cancer Registration and Analysis Service (NCRAS) and patient‐level Index of Multiple Deprivation. The CPRD is the largest primary care database in the world, with CPRD GOLD covering about 7% of UK population, and being representative of the English population.^10^

The linked dataset was derived from a larger linked dataset consisting also of secondary data from Hospital Episodes Statistics (HES) and of 11 common cancer sites (CPRD Independent Scientific Advisory Committee approved, Protocol 17_107). Bladder and renal cancer patients aged 25 years and over first diagnosed between April 2012 and December 2015 were extracted from CPRD first using Readcodes for cancer diagnosis. Additional bladder and renal cancer cases from NCRAS were then identified from patients with the other nine cancers, using the International Classification of Diseases 10th revision.

Patients with at least one of the examined relevant symptom in the year before diagnosis were included, all of whom had cancer stage and route to diagnosis information.

### 2.2 Dependent variable

Stage of cancer diagnosis was obtained from NCRAS and derived from the tumour, node and metastasis (TNM) staging method. Our analysis outcome was dichotomised into early (TNM Stages 0–2) and advanced stages (TNM Stages 3 and 4), as previously used.^11^

### 2.3 Independent variables

We obtained lists of symptom Readcodes for bladder and renal cancer that were previously used by our collaborators from the Hamilton group.^12^, ^13^ This group was the first UK research group to use CPRD to examine cancer symptoms in primary care, using Readcodes which they collated using robust methods.^14^ Symptom codes were checked and sorted into five relevant groups (haematuria, UTI, abdominal symptoms, systemic symptoms and other urogenital symptoms) by a practising general practitioner (GP) (Y.Z.). These categories were defined based on their associations with urological cancers (haematuria and UTI), anatomical (abdominal symptoms and urogenital symptoms) or clinical relevance (systemic symptoms). In line with previous studies examining symptoms before cancer diagnosis, we defined the presenting symptom as the first relevant symptom or UTI diagnosis in the year before cancer diagnosis,^9^ as this is the most relevant period for a symptom to be attributable to the subsequently diagnosed cancer. Patients were assigned a ‘yes/no’ for each relevant symptom examined. In patients who had multiple relevant symptoms on the same presenting date, they were classified as the “multiple symptoms” group. The variable ‘presenting symptom’ therefore contained patients with exclusively one of the five symptom groups examined or multiple symptoms. Although UTI itself is not a symptom, clinical codes containing symptoms, diagnosis and positive urine dipstick suggestive of UTIs (i.e. positive leukocytes and/or nitrites) were all included under the symptom group of ‘UTI’. We did so as most UTIs diagnosed in general practice are presumptive, and the same clinical codes may reflect either symptoms or diagnosis of UTI.

Patient sex was obtained from CPRD. Age at diagnosis was estimated using the difference between the year of cancer diagnosis (obtained from NCRAS) and the patient's date of birth using the mid‐point of a calendar year (1st July), as only the year of birth is provided by CPRD GOLD. Ten‐year age groups were created between 45 and 85 years old. ‘Route to diagnosis’ was obtained from NCRAS. Patient‐level deprivation was provided at source through the linked patient‐level Index of Multiple Deprivation score, which was used to stratify patients into deprivation quintiles for patients with all 11 cancers.

### 2.4 Data analysis

We presented descriptive statistics for patient characteristics, by cancer type and cancer stage at diagnosis. We investigated associations between stage at diagnosis and presenting symptoms using multivariate logistic regression, where stage was a binary (early/advanced) outcome variable. We adjusted the logistic models with covariates age (groups), sex, deprivation and route to diagnosis.

Due to prior clinical considerations that sex and age might modify the effect of symptoms on cancer stage, or vice versa, we investigated possible interactions between presenting symptoms and age or sex. First, we adjusted for interaction between presenting symptoms and sex, and then separately, for presenting symptoms and age. We derived the marginal effect of the predicted probability of advanced‐stage cancer diagnosis for sex, age and presenting symptoms, respectively, from these models. We also reported the crude effects of the interaction analysis without adjusting for any covariates.

### 2.5 Sensitivity analysis

As diagnostic intervals can affect the association between presenting symptoms and stage, we repeated the main analysis for patients with a total diagnostic interval (from presenting symptom to diagnosis date) of up to and including (a) 60 and (b) 90 days.

We performed all analyses separately for bladder and renal cancer patients, using Stata SE, version 17.0.

## 3 RESULTS

About 51% (*n* = 5113) of the bladder and renal cancer patients in our CPRD cohort had linkage to NCRAS. Of the 2634 patients in the linked dataset, 1591 (60.4%) had at least one relevant symptom recorded in the year before cancer diagnosis. We therefore analysed data from 1151 bladder cancer and 440 renal cancer patients.

42 (3.6%) bladder cancer and 14 (3.2%) renal cancer patients had more than one symptom at presentation, with the maximum number of presenting symptoms being three (*n* = 5). The highest proportions of advanced‐stage bladder (34.4%) and renal (32.2%) cancer were in patients aged 65–74 years old (Table 1). Higher proportions of women were diagnosed at advanced (41.7%) than early stage (23.4%) for bladder cancer, but this rate was similar in renal cancer. For bladder cancer, haematuria was more likely in early than in advanced‐stage cancer patients (46.1% vs. 29.1% for early vs. late diagnosis), while UTI and abdominal symptoms were more likely in advanced than in early‐stage cancer. For renal cancer, proportions of presenting symptoms were similar between early and advanced cases (Table 1).

**TABLE 1:** Baseline characteristics of bladder and renal cancer patients, stratified by stages of diagnosis.

Variable	Bladder cancer			Renal cancer		
	Total	Early stage	Late stage	Total	Early stage	Late stage
	*N*	*N* (%)	*N* (%)	*N*	*N* (%)	*N* (%)
Age (years)						
Median (IQR)	74 (67–80)	74 (67–80)	74 (67–80)	68 (59–76)	66 (57–74)	70 (61–78)
<45	29 (2.5)	25 (2.5)	4 (2.6)	24 (5.5)	17 (8.6)	7 (2.9)
45–54	61 (5.3)	52 (5.2)	9 (6.0)	56 (12.7)	24 (12.1)	32 (13.2)
55–64	151 (13.1)	134 (13.4)	17 (11.3)	90 (20.5)	49 (24.7)	41 (16.9)
65–74	387 (33.6)	335 (33.5)	52 (34.4)	142 (32.3)	64 (32.3)	78 (32.2)
75–84	383 (33.3)	334 (33.4)	49 (32.5)	100 (22.7)	32 (16.2)	68 (28.1)
85+	140 (12.2)	120 (12.0)	20 (13.2)	28 (6.4)	12 (6.1)	16 (6.6)
Sex						
Male	854 (74.2)	766 (76.6)	88 (58.3)	278 (63.2)	124 (62.6)	154 (63.6)
Female	297 (25.8)	234 (23.4)	63 (41.7)	162 (36.8)	74 (37.4)	88 (36.4)
Deprivation quintile						
1 (highest)	276 (24.0)	245 (24.5)	31 (20.5)	104 (23.6)	47 (23.7)	57 (23.6)
2	275 (23.9)	232 (23.2)	43 (28.5)	107 (24.3)	45 (22.7)	62 (25.6)
3	239 (20.8)	208 (20.8)	31 (20.5)	95 (21.6)	40 (20.2)	55 (22.7)
4	216 (18.8)	191 (19.1)	25 (16.6)	78 (17.7)	35 (17.7)	43 (17.8)
5 (lowest)	145 (12.6)	124 (12.4)	21 (13.9)	56 (12.7)	31 (15.7)	25 (10.3)
Route to diagnosis						
Routine GP referral	415 (36.1)	385 (38.5)	30 (26.1)	121 (27.5)	56 (28.3)	65 (26.9)
Fast‐track GP referral	481 (41.8)	414 (41.4)	67 (44.4)	161 (36.6)	77 (38.9)	84 (34.7)
Emergency presentation	100 (8.7)	64 (6.4)	36 (23.8)	79 (18.0)	20 (10.1)	59 (24.4)
Inpatient elective	12 (1.0)	11 (1.1)	1 (1.2)	6 (1.4)	4 (2.0)	2 (0.8)
Other outpatient	122 (10.6)	108 (10.8)	14 (10.9)	61 (13.9)	36 (18.2)	25 (10.3)
Unknown	21 (1.8)	18 (1.8)	3 (2.9)	12 (2.7)	5 (2.5)	7 (2.9)
Presenting symptoms						
Haematuria	505 (43.9)	461 (46.1)	44 (29.1)	85 (19.3)	34 (17.3)	51 (21.0)
UTI	188 (16.3)	151 (15.1)	37 (24.5)	40 (9.1)	21 (10.7)	19 (7.8)
Abdominal symptoms^a^	170 (14.8)	138 (13.8)	32 (21.2)	141 (32.0)	64 (32.5)	77 (31.7)
Abdominal pain	117 (10.2)	97 (9.7)	20 (13.2)	95 (21.6)	35 (17.8)	60 (24.7)
Loin pain	9 (0.8)	6 (0.6)	3 (2.0)	18 (4.1)	11 (5.6)	7 (2.9)
Bowel symptoms	44 (3.8)	35 (3.5)	9 (6.0)	28 (6.4)	18 (9.1)	10 (4.1)
Systemic symptoms^a^	205 (17.8)	176 (17.6)	29 (19.2)	137 (31.1)	60 (30.5)	77 (31.7)
Fever	3 (0.3)	2 (0.2)	1 (0.7)	3 (0.7)	1 (0.5)	2 (0.8)
Non‐specific symptoms^b^	44 (3.8)	35 (3.5)	9 (6.0)	35 (8.0)	14 (7.1)	21 (8.6)
Acute systemic symptom^c^	4 (0.3)	3 (0.3)	1 (0.7)	6 (1.4)	1 (0.5)	5 (2.1)
Anaemia	14 (1.2)	12 (1.2)	2 (1.3)	18 (4.1)	5 (2.5)	13 (5.3)
Hypertension	140 (12.2)	124 (12.4)	16 (10.6)	75 (17.0)	39 (19.8)	36 (14.8)
Other urogenital symptoms^a^	41 (3.6)	38 (3.8)	3 (2.0)	23 (5.2)	11 (5.6)	12 (4.9)
Other urinary symptoms^d^	29 (2.5)	26 (2.6)	3 (2.0)	15 (3.4)	8 (4.1)	7 (2.9)
Genital itch	12 (1.0)	12 (1.2)	0 (0.0)	8 (1.8)	3 (1.5)	5 (2.1)
Multiple symptoms	42 (3.6)	36 (3.6)	6 (4.0)	14 (3.2)	7 (3.6)	7 (2.9)

^a^ Symptoms include subcategories that are collapsed into main symptom groups in analyses.

^b^ Non‐specific symptoms include weight loss, loss of appetite, fatigue, weakness and leg swelling.

^c^ Acute systemic symptoms: confusion and vomiting.

^d^ Other urinary symptoms: nocturia, poor urinary stream, urinary incontinence and retention.

### 3.1 Main effect analysis

In adjusted analyses, sex, route to diagnosis and presenting symptom with advanced bladder cancer (Table 2). Women were more likely to have advanced‐stage diagnosis than men (OR 1.89, CI 1.29–2.78; *p* = 0.001). Emergency presentation and GP fast‐track referral routes were associated with a seven‐ and two‐fold greater odds of advanced stage, respectively (OR 7.02, CI 3.93–12.51 and OR 2.23, CI 1.40–3.55), compared with a routine GP referral. Considering presenting symptoms, there was evidence for increased odds of advanced bladder cancer in patients that presented with UTIs (OR 2.22, CI 1.34–3.69) and abdominal symptoms (OR 2.19, CI 1.30–3.70), compared with those that presented with haematuria.

**TABLE 2:** Association between patient factors, route to diagnosis and symptoms and advanced stage of cancer diagnosis.

Variable	Bladder cancer		Renal cancer	
	OR (95% CI) for advanced stage	*p* value	OR (95% CI) for advanced stage	*p* value
Age group				
<45	‐	0.911	0.69 (0.11, 4.29)	0.135
45–54	1.19 (0.52, 2.72)		1.30 (0.68, 2.47)	
55–64	Reference		Reference	
65–74	1.09 (0.59, 2.01)		1.40 (0.80, 2.44)	
75–84	1.05 (0.57, 1.94)		2.35 (1.26, 4.37)	
85+	0.83 (0.40, 1.75)		1.19 (0.48, 2.92)	
Sex				
Male	Reference	0.001	Reference	0.985
Female	1.89 (1.29, 2.78)		1.00 (0.66, 1.52)	
Deprivation quintile				
1	Reference	0.266	Reference	0.581
2	1.60 (0.95, 2.70)		1.19 (0.67, 2.10)	
3	1.24 (0.71, 2.17)		0.98 (0.54, 1.78)	
4	0.90 (0.50, 1.63)		1.03 (0.55, 1.94)	
5	1.12 (0.60, 2.11)		0.65 (0.32, 1.32)	
Route to diagnosis				
Routine GP	Reference	<0.001	Reference	0.004
Fast‐track GP	2.23 (1.40, 3.55)		0.92 (0.56, 1.52)	
Emergency presentation	7.02 (3.93, 12.51)		2.62 (1.37, 5.03)	
Inpatient elective	1.00 (0.12, 8.24)		0.44 (0.07, 2.71)	
Other outpatient	1.62 (0.81, 3.22)		0.58 (0.31, 1.10)	
Unknown	2.07 (0.56, 7.66)		1.30 (0.38, 4.48)	
Presenting symptoms				
Haematuria	Reference	0.016	Reference	0.593
UTI	2.22 (1.34, 3.69)		0.50 (0.22, 1.14)	
Abdominal symptoms	2.19 (1.30, 3.70)		0.72 (0.40, 1.30)	
Systemic symptoms	1.50 (0.88, 2.53)		0.74 (0.42, 1.34)	
Other urogenital symptoms	0.86 (0.24, 3.02)		0.58 (0.22, 1.55)	
Multiple symptoms	1.35 (0.50, 3.61)		0.48 (0.14, 1.65)	
Multiple symptoms	1.35 (0.50, 3.61)		0.48 (0.14, 1.65)	

Patients diagnosed through emergency presentation had a two‐fold likelihood of an advanced‐stage diagnosis compared with those diagnosed via a routine GP referral (OR 2.62, CI 1.37–5.03) (Table 2). There were no statistically signification associations between age, sex, deprivation and presenting symptoms and stage at diagnosis, possibly due to small sample size.

### 3.2 Interaction analysis

**FIGURE 1:**
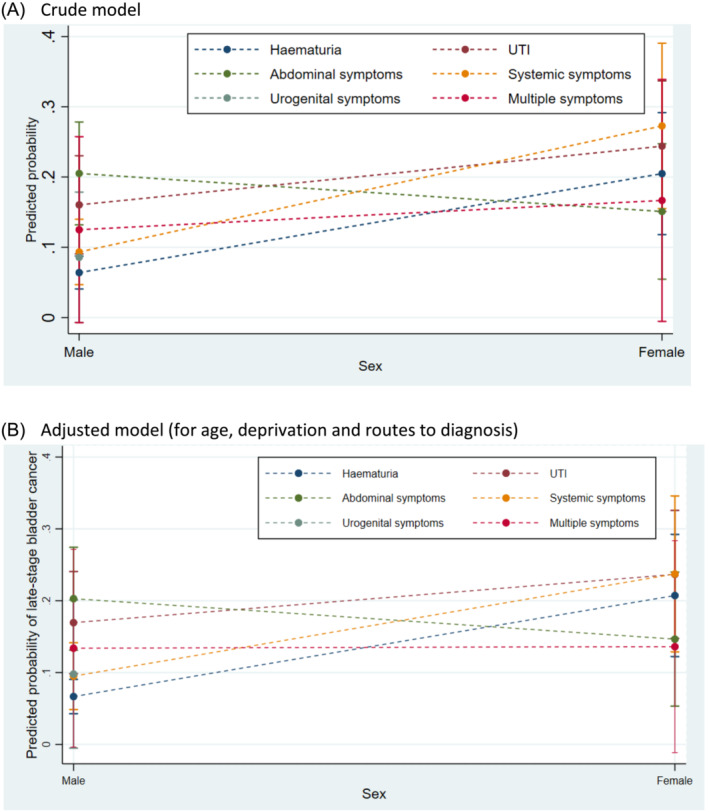
Predicted probability of advanced‐stage bladder cancer diagnosis by sex and presenting symptoms.

There was evidence for an interaction between sex and presenting symptoms for bladder cancer (*p* = 0.023, Figure 1A,B). The main effect for women versus men was 1.89 (CI 1.29, 2.78, *p* = 0.001; Table 2) before including an interaction term between sex and presenting symptoms, and this became 1.02 (CI 0.16–6.30, *p* = 0.023; results not shown) after adjusting for sex–symptom interaction. This suggests that differences in symptom presentation between men and women might explain some of the sex inequality seen with respect to advanced‐stage bladder cancer.

Considering haematuria, the proportion of women with advanced‐stage bladder cancer was similar in those who presented with and without haematuria (20.5% and 21.5%, respectively). However, the proportion of men who had advanced‐stage bladder cancer was 6.4% and 14.1% in those who presented with and without haematuria, respectively, suggesting that haematuria was a protective factor for advanced‐stage bladder cancer in men (Appendix S1). In contrast, the proportion of men with advanced‐stage bladder cancer is higher in those presenting with UTIs (16.0% vs. 9.5%) or abdominal symptoms (20.5% vs. 8.7%) compared to those without the respective symptoms.

Men with abdominal symptoms had higher predicted probability of advanced‐stage bladder cancer than women (20% vs. 15%; Figure 1A,B, Appendix S2A). Comparing presenting symptoms, the sex inequality for advanced disease was the greatest for women with haematuria (21% vs. 7%, women vs. men) and systemic symptoms (24% vs. 10%, women vs. men, respectively, Figure 1A,B; Appendix S2A).

Although there was an increased probability of advanced‐stage renal cancer in women presenting with multiple symptoms, haematuria and UTI compared to men, the numbers in the symptom–advanced‐stage strata were small and results were statistically insignificant (Appendix S2B).

There was no significant interaction between age group and presenting symptoms, for patients with bladder and renal cancer (Appendix S3).

### 3.3 Sensitivity analysis

For bladder cancer, sensitivity analysis performed on patients with a diagnostic interval of up to 90 and 60 days showed a similar effect size and direction on advanced‐stage diagnosis for sex, emergency presentation, UTI and abdominal symptoms (Appendix S4). There were no statistically significant associations between all examined independent variables and stage at diagnosis for renal cancer in the sensitivity analyses.

## 4 DISCUSSION

We found evidence that women, patients diagnosed following an emergency presentation or a GP fast‐track referral, presenting with UTIs or abdominal symptoms were associated with increased odds of advanced‐stage bladder cancer. Sex differences in presenting symptoms may partially explain the increased likelihood of advanced‐stage bladder cancer in women than men. The greatest sex inequality of advanced‐stage bladder cancer was seen in patients with haematuria. We focused our discussions on bladder cancer subsequently as the study was underpowered for renal cancer due to smaller sample in this group of patients.

### 4.1 Comparing with previous literature

Our findings are in line with existing evidence that women were more likely than men to be diagnosed with advanced‐stage bladder cancer^11^, ^15^, ^16^ and that bladder cancer patients presenting with UTIs were more likely to have worse outcomes than those presenting with haematuria.^2^, ^17^, ^18^ Additionally, we also examined the effect of not only UTI but also a broader range of symptoms on stage of diagnosis. We also further characterised the effect of differences in presenting symptoms between sexes on advanced‐stage bladder cancer diagnosis.

With respect to routes to diagnosis, we found strong associations for being diagnosed through an emergency and advanced‐stage diagnosis. This is in line with existing evidence that patients diagnosed through this route often have advanced‐stage cancer and worse survival.^19^

### 4.2 Strengths and limitations

To our knowledge, our study is the first to use a national cohort of bladder and kidney cancer patients to examine the contribution of symptoms and sex on stage at diagnosis. The linked data allowed detailed examination of presenting symptoms pre‐diagnosis, enriching our understanding of the potential mechanisms contributing to sex differences in cancer stage at diagnosis in symptomatic patients.

Our study used coded information which may be subject to variations in clinician coding behaviours. However, the CPRD represents one of the best primary care data sources available worldwide and has been used extensively for examining symptomatic presentations of different cancers, including for bladder and kidney cancer.^9^, ^12^, ^13^, ^20^, ^21^ A further limitation is that although 1 year is likely to be the most relevant period for an associated bladder and renal cancer symptom to present to primary care,^21^ symptoms might also occur more than 12 months pre‐diagnosis.

For renal cancer cases, although we observe an effect size in some independent variables with cancer stage, we cannot confirm the statistical significance due to small numbers in each association stratum. Therefore, further investigations in larger samples are needed.

### 4.3 Interpretations and implications

Our study suggests that presenting symptoms and sex–symptom interactions both explain the sex inequality observed in stage at diagnosis in patients with bladder cancer. Of all symptoms examined, the largest sex disparity in advanced‐stage diagnosis was seen in patients presenting with haematuria. The reasons for this observation may be two‐fold. First, 49.4% and 27.9% of men and women presented with haematuria respectively, with higher proportion of men presenting with the symptom associated with the lowest likelihood of advanced bladder cancer. Therefore, some of the sex inequality seen in bladder stage at diagnosis may be due to biological differences between men and women. Next, the interaction analysis suggests that presenting with haematuria is a protective factor for advanced‐stage bladder cancer in men, more so than in women. This may reflect an underlying biological difference in how early in the disease process bladder tumours bleed in men compared with women and/or sex variation in the length of time intervals from symptom to help‐seeking and from help‐seeking to referral and specialist investigation. Improving risk stratification of women with urological symptoms, including haematuria, is justified, as it may reduce some of the avoidable causes of advanced bladder cancer seen in women.

In our study, men with abdominal symptoms were more likely to have advanced‐stage bladder cancer than women with abdominal symptoms. This may be due to clinicians being more likely to investigate women for gynaecological causes of abdominal symptoms, which may lead to an eventual bladder cancer diagnosis. A Danish study found that besides gynaecological cancers, bladder cancer was the only other abdominal cancer in which the incidence rate of transvaginal ultrasound use increased in the 4 months preceding the cancer diagnosis, supporting this hypothesis.^22^ Second, men with abdominal symptoms may be presenting later than women with these symptoms. Existing evidence suggest that men delay help‐seeking in general due to psychosocial factors, such as poor understanding and normalisation of symptoms, embarrassment or fear and conformity to masculine gender role norms.^23^, ^24^, ^25^ Therefore, they may first present with more progressive disease than women, resulting in advanced‐stage diagnosis. Nevertheless, it is important to note that although there is a sex difference in odds of advanced‐stage bladder cancer in patients with abdominal symptoms, the disparity is small and should be interpreted with caution.

In line with existing literature, patients with UTIs were more likely to have worse stage at diagnosis than those with haematuria. Despite previous suggestions that women with UTIs were particularly at risk of worse outcomes, presenting with UTI increases the risk of advanced bladder cancer in both men and women. It is possible that clinicians are equally likely to attribute UTIs to a benign cause in men and women. Quantifying cancer risk in subgroups of patients with UTIs (such as defining number of episodes and periodicity of UTIs which may represent higher cancer risk) may help with risk stratification and guiding appropriate referrals in these patients.^26^

### 4.4 Conclusion

We found evidence for increased odds of advanced‐stage bladder cancer in patients presenting with UTIs and abdominal symptoms, compared to those with haematuria. Among the examined symptoms, men presenting with haematuria were the most protected from advanced bladder cancer. Conversely, men with abdominal symptoms were more likely to be diagnosed with advanced‐stage bladder cancer than women with the same symptoms. Our findings suggest that future research to quantify cancer risk and identify higher risk women with haematuria, and in subgroups of patients with UTIs, may improve the diagnostic process for these patients and potentially reduce the sex inequality observed in advanced‐stage bladder cancer diagnosis.

## AUTHOR CONTRIBUTIONS

**Yin Zhou**: Conceptualisation; methodology; formal analysis; writing—original draft; writing—review and editing; funding acquisition. **Georgios Lyratzopoulos**: Conceptualisation; methodology; formal analysis; writing—review and editing; supervision. **Prabhakar Rajan**: Writing—review and editing. **Fiona M. Walter**: Conceptualisation; writing—review and editing; supervision. **Jianhua Wu**: Methodology; formal analysis; writing—original draft; writing—review and editing; supervision.

## CONFLICT OF INTEREST STATEMENT

The authors declare no conflict of interest.

## CONSENT FOR PUBLICATION

No identifiable patient information was used in this study.

## Supporting information


**Data S1.** Supporting Information

**Data S2.** Supporting Information

**Appendix S1:** Proportion of patients with advanced‐stage bladder cancer by symptoms and sex.
**Appendix S2:** Crude and adjusted predicted probabilities for advanced‐stage cancer by sex and symptoms.
**Appendix S3:** Adjusted predicted probability of advanced‐stage cancer diagnosis by age group and presenting symptoms.
**Appendix S4:** Sensitivity analyses – bladder cancer.

## Data Availability

The original data may be accessible through contact with the research team and subject to approval from CPRD.
