# Supplementary material for: Understanding symptom contribution to sex inequality in bladder and renal cancer stage at diagnosis
Source: BJUI Compass. 2024 Apr 19;5(7):691–8. doi: 10.1002/bco2.360 (PMC11249815; doi:10.1002/bco2.360)
Supplement: Supplementary file 3 — Appendix S1: Proportion of patients with advanced‐stage bladder cancer by symptoms and sex. Appendix S2: Crude and adjusted predicted probabilities for advanced‐stage cancer by sex and symptoms. Appendix S3: Adjusted predicted probability of advanced‐stage cancer diagnosis by age group and presenting symptoms. Appendix S4: Sensitivity analyses – bladder cancer. [file BCO2-5-691-s002.pdf]

Appendix 1: Proportion of patients with advanced-stage bladder cancer by symptoms and sex

**Presenting symptoms**	**Male**		**Female**	
	**Total**	**Number of advanced-stage cancer (%)**	**Total**	**Number of advanced-stage cancer (%)**
Overall	854	88 (10.3)	297	63 (21.2)
Haematuria				
Yes	422	27 (6.4)	83	17 (20.5)
No	432	61 (14.1)	214	46 (21.5)
Urinary tract infections				
Yes	106	17 (16.0)	82	20 (24.4)
No	748	71 (9.5)	215	43 (20.0)
Systemic symptoms				
Yes	150	14 (9.3)	55	15 (27.3)
No	704	74 (10.5)	242	48 (19.8)
Abdominal symptoms				
Yes	117	24 (20.5)	53	8 (15.1)
No	737	64 (8.7)	244	55 (22.5)
Urogenital symptoms				
Yes	35	3 (8.6)	6	0
No	819	85 (10.4)	291	63 (21.6)
Multiple symptoms				
Yes	24	3 (12.5)	18	3 (16.7)
No	830	85 (10.2)	279	60 (21.5)

Appendix 2: Crude and adjusted predicted probabilities for advanced-stage cancer by sex and symptoms

A: Bladder cancer

Presenting symptoms	Crude predicted probabilities for advanced-stage bladder cancer (95% CI)		Adjusted predicted probabilities for advanced-stage bladder cancer (95% CI)	
	Male	Female	Male	Female
Haematuria	0.06 (0.04 – 0.09)	0.20 (0.12 – 0.29)	0.07 (0.04 – 0.09)	0.21 (0.12 – 0.29)
UTI	0.16 (0.09 – 0.23)	0.24 (0.15 – 0.34)	0.17 (0.10 – 0.24)	0.24 (0.15 – 0.33)
Abdominal symptoms	0.21 (0.13 – 0.28)	0.15 (0.05 – 0.25)	0.20 (0.13 – 0.27)	0.15 (0.05 – 0.24)
Systemic symptoms	0.09 (0.05 – 0.14)	0.27 (0.16 – 0.39)	0.10 (0.05 – 0.14)	0.24 (0.13 – 0.35)
Urogenital symptoms	0.09 (-0.01 – 0.18)	-	0.10 (-0.01 – 0.20)	-
Multiple symptoms	0.13 (-0.01 – 0.26)	0.17 (-0.01 – 0.34)	0.10 (0.00 – 0.27)	0.14 (-0.01 – 0.28)

B: Renal cancer

Presenting symptoms	Crude predicted probabilities for advanced-stage bladder cancer (95% CI)		Adjusted predicted probabilities for advanced-stage bladder cancer (95% CI)	
	Male	Female	Male	Female
Haematuria	0.58 (0.46 – 0.69)	0.68 (0.48 – 0.89)	0.59 (0.48 – 0.71)	0.71 (0.52 – 0.91)
UTI	0.37 (0.15 – 0.59)	0.57 (0.36 – 0.78)	0.37 (0.16 – 0.58)	0.55 (0.34 – 0.75)
Abdominal symptoms	0.61 (0.51 – 0.72)	0.46 (0.33 – 0.58)	0.60 (0.50 – 0.71)	0.47 (0.35 – 0.59)
Systemic symptoms	0.53 (0.43 – 0.64)	0.59 (0.45 – 0.73)	0.54 (0.44 – 0.64)	0.58 (0.44 – 0.71)
Urogenital symptoms	0.56 (0.32 – 0.81)	0.43 (0.06 – 0.80)	0.54 (0.30 – 0.78)	0.39 (0.04 – 0.74)
Multiple symptoms	0.44 (0.12 – 0.77)	0.60 (0.17 – 1.03)	0.34 (0.04 – 0.64)	0.63 (0.22 – 1.04)

Appendix 3: Adjusted predicted probability of advanced-stage cancer diagnosis by age group and presenting symptoms

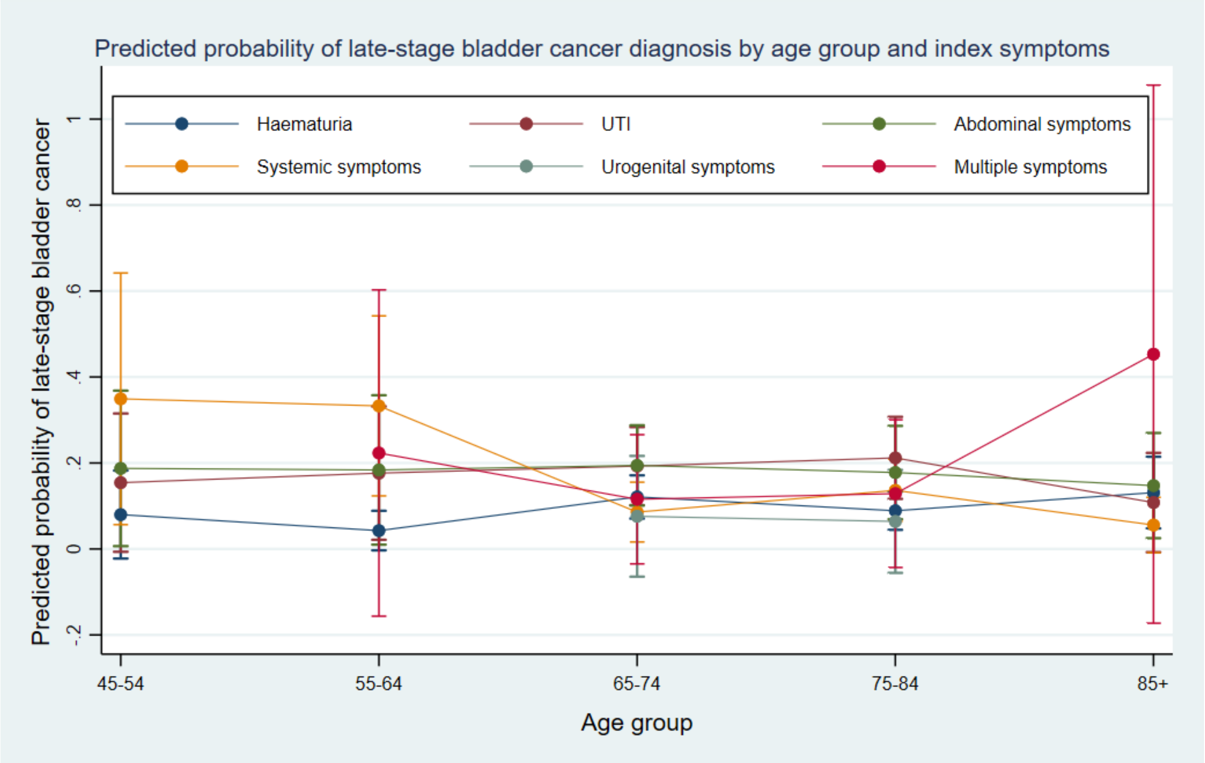

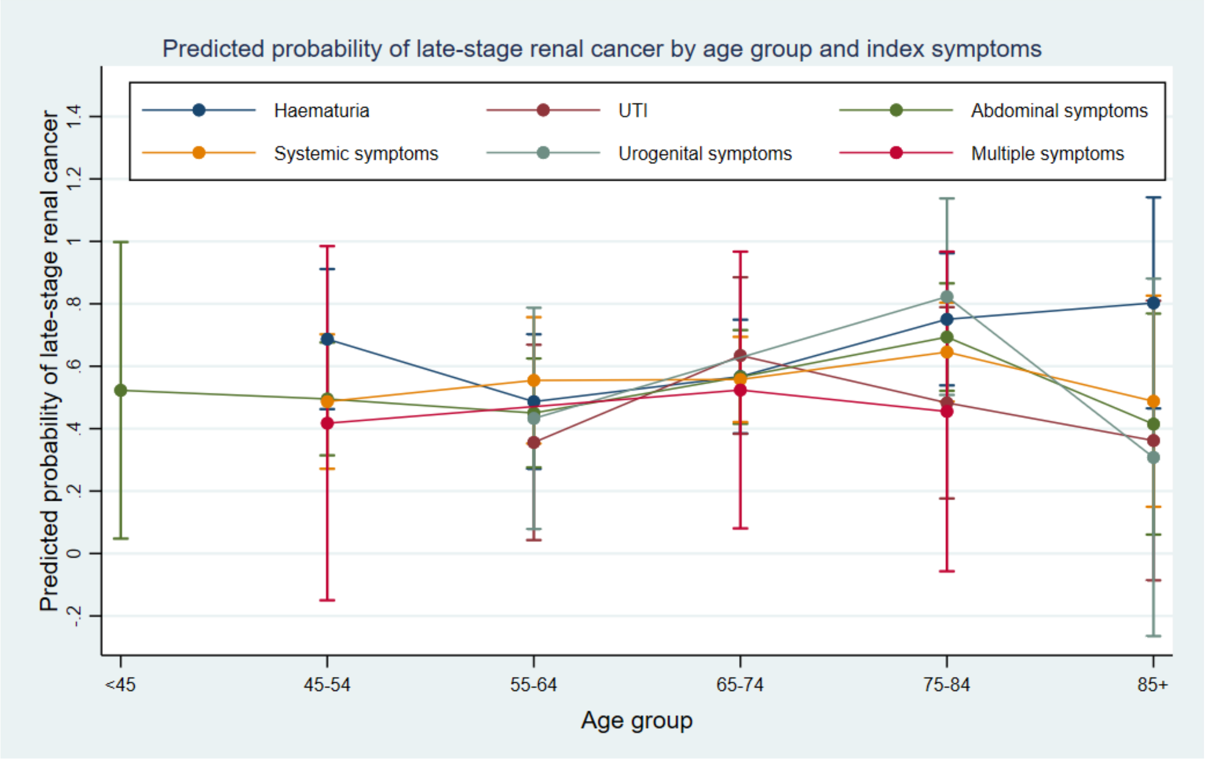

Appendix 4: Sensitivity analyses – bladder cancer

Variable	Total diagnostic interval <91 days				Total diagnostic interval <61 days			
	Bladder cancer		Renal cancer		Bladder cancer		Renal cancer	
	OR (95% CI)	p-value	OR (95% CI)	p-value	OR (95% CI)	p-value	OR (95% CI)	p-value
**Age group**								
<45	-	0.884	-	0.284	-	0.970	-	0.187
45-54	1.01 (0.28, 3.63)		2.95 (0.82, 10.63)		0.95 (0.22, 4.01)		5.79 (0.86, 39.26)	
55-64	Reference		Reference		Reference		Reference	
65-74	1.43 (0.59, 3.48)		1.35 (0.46, 3.98)		1.30 (0.49, 3.44)		1.12 (0.29, 4.35)	
75-84	1.07 (0.42, 2.71)		2.89 (0.82, 10.18)		1.07 (0.39, 2.92)		3.61 (0.69, 18.96)	
85+	1.06 (0.32, 3.44)		1.14 (0.18, 7.04)		1.26 (0.34, 4.66)		3.26 (0.23, 46.99)	
**Sex**								
Male	Reference	0.014		0.904	Reference	0.034	Reference	0.228
Female	2.24 (1.18, 4.27)		0.95 (0.41, 2.20)		2.20 (1.06, 4.54)		0.47 (0.14, 1.60)	
**Deprivation quintile**								
1	Reference	0.666	Reference	0.890	Reference	0.825	Reference	0.992
2	1.01 (0.46, 2.23)		1.01 (0.35, 2.88)		1.33 (0.57, 3.12)		0.71 (0.16, 3.11)	
3	0.87 (0.37, 2.06)		1.24 (0.41, 3.78)		0.90 (0.34, 2.34)		0.90 (0.22, 3.78)	
4	0.58 (0.24, 1.40)		1.63 (0.42, 6.37)		0.76 (0.30, 1.91)		0.76 (0.11, 5.43)	
5	0.61 (0.22, 1.71)		0.76 (0.18, 3.18)		0.94 (0.31, 2.85)		0.74 (0.08, 6.83)	
**Route to diagnosis**								
Routine GP	Reference	<0.001	Reference	0.432	Reference	<0.001	Reference	0.616
TWW	2.44 (1.07, 5.52)		0.51 (0.18, 1.45)		1.83 (0.75, 4.43)		0.36 (0.08, 1.62)	
Emergency presentation	12.61 (4.61, 34.49)		1.64 (0.42, 6.45)		10.30 (3.41, 31.17)		1.04 (0.18, 6.12)	
Inpatient elective	2.00 (0.15, 26.11)		0.23 (0.01, 3.53)		1.56 (0.11, 21.19)		0.24 (0.01, 5.03)	
Other outpatient	1.41 (0.42, 4.69)		0.78 (0.14, 4.20)		1.34 (0.38, 4.72)		1.43 (0.11, 18.85)	
Unknown	1.00 (0.00, 0.00)		0.52 (0.09, 3.03)		-		0.65 (0.07, 6.45)	
**Presenting symptoms**								

Haematuria	Reference	0.035	Reference	0.659	Reference	0.045	Reference	0.372
UTI	2.20 (1.01, 4.77)		0.91 (0.07, 11.21)		2.29 (0.93, 5.67)		-	
Abdominal symptoms	2.70 (0.98, 7.42)		0.57 (0.23, 1.41)		2.89 (0.95, 8.80)		1.15 (0.32, 4.16)	
Systemic symptoms	1.05 (0.26, 4.30)		0.95 (0.31, 2.95)		0.85 (0.14, 5.24)		4.26 (0.65, 27.91)	
Other urogenital symptoms	6.38 (1.33, 30.59)		1.00 (0.00, 0.00)		7.06 (1.41, 35.32)		-	
Multiple symptoms	0.41 (0.04, 3.88)		0.30 (0.03, 2.79)		0.48 (0.05, 4.84)		0.48 (0.04, 5.03)	
